# Rare Association Between Atrioventricular Septal Defect and Partial Anomalous Pulmonary Venous Connection

**DOI:** 10.21470/1678-9741-2019-0203

**Published:** 2019

**Authors:** Alexandre Noboru Murakami, Gabriela Guimarães Baston, Mariana Ribeiro Rodero Cardoso, Carlos Henrique De Marchi, Ulisses Alexandre Croti

**Affiliations:** 1 Serviço de Cirurgia Cardíaca do Norte do Paraná, Universidade Estadual de Londrina (UEL), Londrina, PR, Brazil.; 2 Pediatric Cardiovascular Surgery Department of Hospital da Criança e Maternidade de São José do Rio Preto (FUNFARME), Faculdade de Medicina de São José do Rio Preto (FAMERP), SP, Brazil.

**Keywords:** Heart Septal Defects, Atrioventricular Septal Defect, Pulmonary Veins, Cardiopulmonary Bypass

## Abstract

**Clinical data:**

Infant, 7 months, female, referred to our department at one month of age, suspecting of congenital heart disease for further investigation.

**Chest radiography:**

Demonstrates cardiomegaly and prominent pulmonary vascular markings.

**Electrocardiography:**

Shows right ventricular hypertrophy and left anterior fascicular block.

**Echocardiography:**

Evidenced common atrioventricular valve with two orifices and the left superior pulmonary vein draining on the brachiocephalic vein.

**Computed tomography angiography:**

This complementary imaging exam was performed to confirm the diagnosis.

**Diagnosis:**

The patient presented an association between AVSD and PAPVC, a rare combination. The clinical picture of heart failure was preponderant, characterized by need for diuretics and complementary exams findings, and early surgical treatment was indicated.

**Operation:**

The operation was performed through a median sternotomy with 123 minutes of cardiopulmonary bypass and 89 minutes of cross-clamping time. The patient had no postoperative complications, remaining 10 days hospitalized.

**Table:** 

Abbreviations, acronyms & symbols	
AV	= Atrioventricular
AVSD	= Atrioventricular septal defect
PAPVC	= Partial anomalous pulmonary venous connection
TAPVC	= Total anomalous pulmonary venous connection

## Clinical Data

Infant, 7 months, female, referred to our department at one month of age, suspecting of congenital heart disease due to heart murmur upon physical examination. No other signs or symptoms and no genetic syndrome were observed.

Physical examination revealed good general condition. Presence of systolic murmur 2+/6+ at upper left sternal border. Eupneic and clear lung sounds. No abdominal findings. Symmetrical peripheral pulses.

During outpatient follow-up, presented with dyspnea on exertion, therefore, diuretics were initiated. At 7 months of age, surgical repair was indicated.

## Chest Radiography

**Fig. 1:**
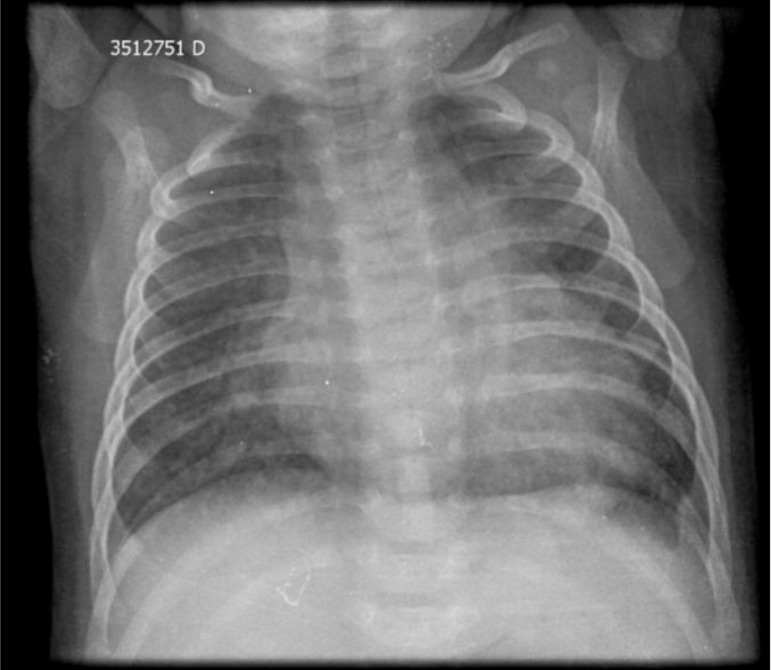
Chest radiography demonstrates cardiomegaly and prominent pulmonary vascular markings.

Posterior-anterior chest radiography shows increased pulmonary vascular markings, more prominent in the upper lungs. Enlarged cardiac area with a cardiothoracic ratio of 0.63 (Figure 1).

## Electrocardiography

**Fig. 2:**
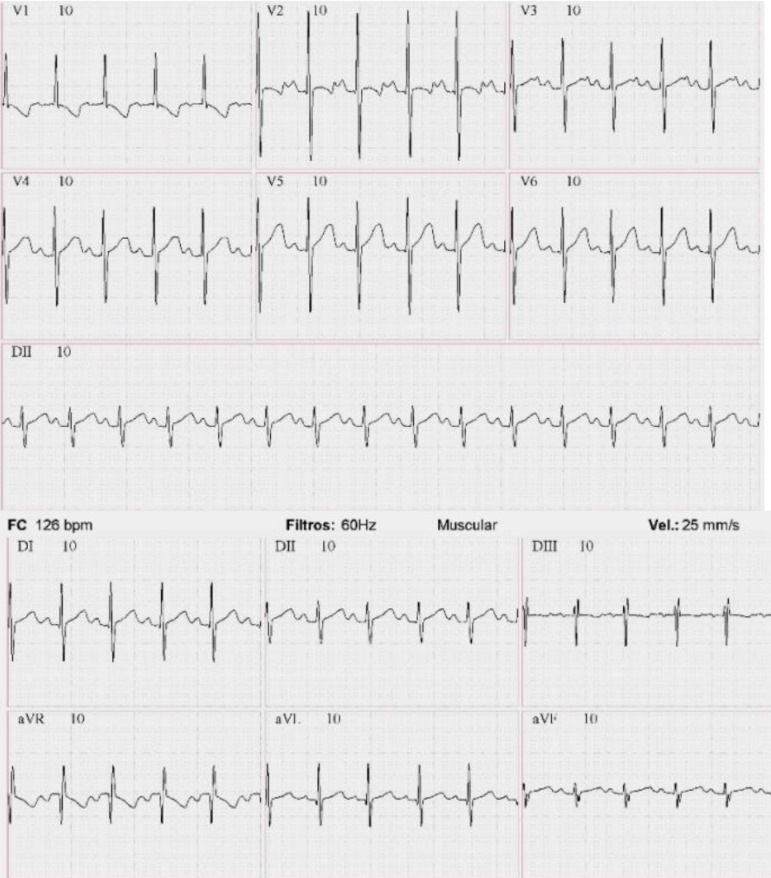
Electrocardiography demonstrates sinus rhythm and right ventricular hypertrophy.

Sinus rhythm, heart rate of 164 beats/min, SAQRS -60º, left anterior fascicular block and right ventricular hypertrophy (Figure 2).

## Echocardiography

*Situs solitus* in levocardia. Abnormal venoatrial and atrioventricular connection and normal ventriculo arterial connection.

Doppler examination demonstrated presence of atrial septal defect (5 mm) with left-to-right shunt and ventricular septal defect (2 mm).

Common atrioventricular valve with two orifices, characterizing a transitional atrioventricular septal defect.

Left superior pulmonary vein draining on the brachiocephalic vein, typical of partial anomalous pulmonary venous connection (PAPVC).

To confirm the diagnosis, the patient underwent a complementary imaging examination.

## Computed tomography angiography

**Fig. 3:**
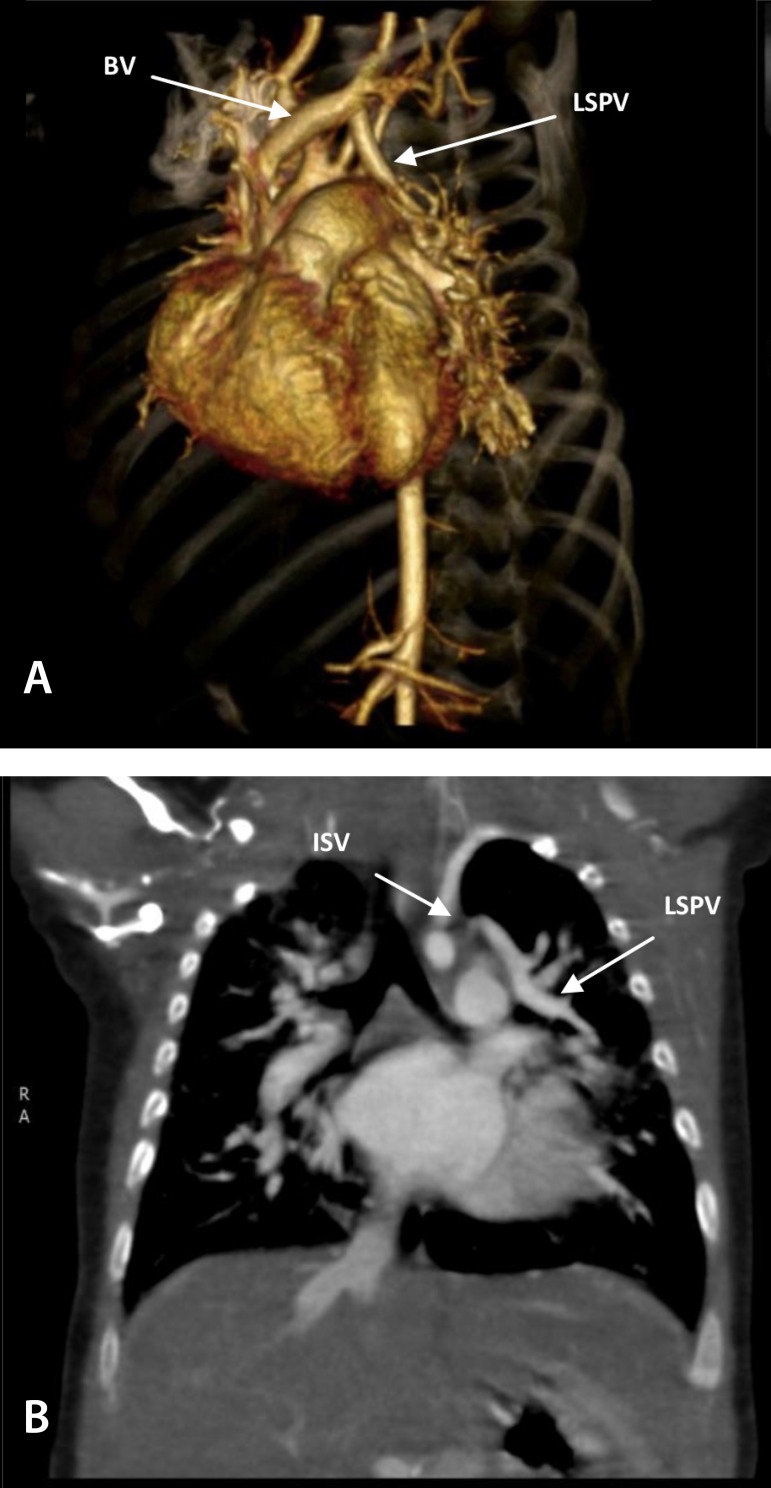
A) 3D volume-rendering computed tomographic angiography showing partial anomalous pulmonary venous connection characterized by LSPV draining directly into BV. B) Small ISV draining in the LSPV before LSPV enters the BV. BV=brachiocephalic vein; ISV=intercostal systemic vein; LSPV=left superior pulmonary vein

Atrioventricular septal defect (AVSD) showing signs of right chamber, pulmonary trunk and left pulmonary artery dilatation. Partial anomalous pulmonary venous return from the upper left lobe in the brachiocephalic vein, as shown in Figure 3.

## Diagnosis

AVSD represents approximately 4% of all cardiac malformations and total anomalous pulmonary venous connection (TAPVC) corresponds to 0.8% of all congenital heart diseases^[1]^. PAPVC was observed in 0.6% to 0.7% of autopsies^[2]^. Presence of left-sided PAPVC is described only in 7% of cases in a series of 236 patients^[2]^. Of the 132 patients who underwent repair of partial AVSD, 62 were associated with other heart defects and the association with PAPVC was not found^[3]^. Another study described an association between anomalous pulmonary venous return and AVSD in 7% of patients^[4]^.

Clinical presentation depends on the type of AVSD. Due to left-to-right shunt at both atrial and ventricular level in complete AVSD and atrioventricular (AV) valve regurgitation, children will develop congestive heart failure^[5]^. Patients with partial AVSD can remain asymptomatic for years.

In this case, the clinical picture of heart failure was preponderant, characterized by need for diuretics and findings of complementary exams.

The echocardiogram performed by an experienced professional was fundamental to identify one of the pulmonary veins connecting anomalously to the brachiocephalic vein and not to the left atrium, which was confirmed by angiography (Figure 3). Computed tomography angiography is the gold standard for the diagnosis of PAPVC and can also evaluate postoperative complications, as demonstrated by Croti et al^[6]^.

The patient presented with a small ventricular septal defect and should have little hemodynamic repercussion, but due to the presence of the anomalous left superior pulmonary vein connection, a greater right-side overload and increased pulmonary flow were observed, and early surgical treatment was indicated.

## Operation

The operation was performed through a median sternotomy with partial thymus resection (left). Heparinization with 4 mg/kg and careful cannulation of the aorta and bicaval were performed as routine. Hypothermia at 28º Celsius with 123 minutes of cardiopulmonary bypass and 89 minutes of cross-clamping time.

**Fig. 4:**
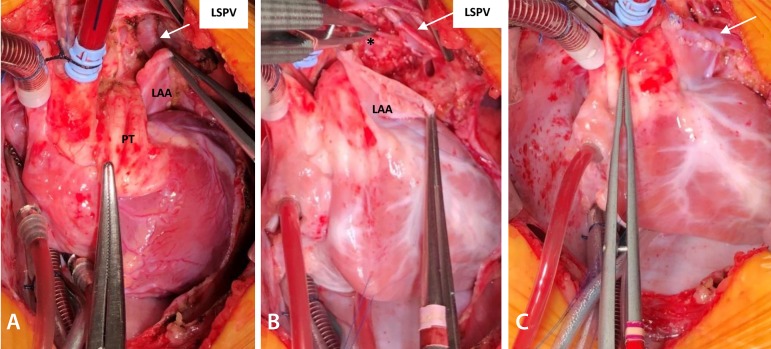
Surgical images. A) Dissected LSPV and its relation to LAA. B) Opened LAA, LSPV ligated previously to being sectioned and systemic vein draining in the LSPV shown by clamp (*). C) Final aspect of the anastomosis between LAA and LSPV with 7-0 polydioxanone suture. LAA=left atrial appendage; LSPV=left superior pulmonary vein; PT=pulmonary trunk

Dissection of left superior pulmonary vein with drainage in the brachiocephalic vein. During dissection, a small intercostal systemic vein was found draining into the pulmonary vein, as shown in angiography (Figure 3B). Ligature and excision of the intercostal vein was performed. Opening on the anterior wall of the pulmonary vein and left atrial appendage and continuous suture with polydioxanone 7-0 between structures (Figure 4).

A common atrioventricular valve with two orifices and a cleft in left AV valve were found. Ventricular septal defect was single-sutured with pledgets, and the cleft in the left AV valve was closed using 6-0 polypropylene sutures. For the atrial septal defect correction, a bovine pericardium patch was used as usual. The patient had no postoperative complications, remaining 10 days hospitalized, including 6 days in a pediatric cardiac care unit and 4 days in the pediatric ward.

**Table:** 

Authors’ roles & responsibilities	
ANM	Substantial contributions to the conception or design of the work; or the acquisition, analysis, or interpretation of data for the work; drafting the work or revising it critically for important intellectual content; final approval of the version to be published
GGB	Substantial contributions to the conception or design of the work; or the acquisition, analysis, or interpretation of data for the work; final approval of the version to be published
MRRC	Substantial contributions to the conception or design of the work; or the acquisition, analysis, or interpretation of data for the work; final approval of the version to be published
CHM	Final approval of the version to be published
UAC	Drafting the work or revising it critically for important intellectual content; final approval of the version to be published
